# Biotechnological approaches to determine the impact of viruses in the energy crop plant *Jatropha curcas*

**DOI:** 10.1186/1743-422X-8-386

**Published:** 2011-08-03

**Authors:** Rose C Ramkat, Alberto Calari, Fatemeh Maghuly, Margit Laimer

**Affiliations:** 1 Plant Biotechnology Unit, IAM, VIBT, BOKU, Muthgasse 18, A - 1190 Vienna, Austria

## Abstract

**Background:**

Geminiviruses infect a wide range of plant species including *Jatropha *and cassava both belonging to family *Euphorbiaceae*. Cassava is traditionally an important food crop in Sub - Saharan countries, while *Jatropha *is considered as valuable biofuel plant with great perspectives in the future.

**Results:**

A total of 127 *Jatropha *samples from Ethiopia and Kenya and 124 cassava samples from Kenya were tested by Enzyme-Linked Immunosorbent Assay (ELISA) for RNA viruses and polymerase chain reaction for geminiviruses. *Jatropha *samples from 4 different districts in Kenya and Ethiopia (analyzed by ELISA) were negative for all three RNA viruses tested: *Cassava brown streak virus *(CBSV), *Cassava common mosaic virus*, *Cucumber mosaic virus*, Three cassava samples from Busia district (Kenya) contained CBSV. Efforts to develop diagnostic approaches allowing reliable pathogen detection in Jatropha, involved the amplification and sequencing of the entire DNA A molecules of 40 Kenyan isolates belonging to *African cassava mosaic virus *(ACMV) and *East African cassava mosaic virus *- *Uganda*. This information enabled the design of novel primers to address different questions: a) primers amplifying longer sequences led to a phylogenetic tree of isolates, allowing some predictions on the evolutionary aspects of Begomoviruses in *Jatrophia*; b) primers amplifying shorter sequences represent a reliable diagnostic tool. This is the first report of the two Begomoviruses in *J. curcas*. Two cassava samples were co - infected with cassava mosaic geminivirus and CBSV. A Defective DNA A of ACMV was found for the first time in *Jatropha*.

**Conclusion:**

Cassava geminiviruses occurring in *Jatropha *might be spread wider than anticipated. If not taken care of, this virus infection might negatively impact large scale plantations for biofuel production. Being hosts for similar pathogens, the planting vicinity of the two crop plants needs to be handled carefully.

## Introduction

Geminiviruses are a group of plant viruses that contain circular single stranded (ss) DNA genomes encapsidated in small twinned icosahedral capsids [1]. They infect a wide range of plant species and are responsible for considerable crop losses [2]. Members of this virus family have been classified into four genera: *Begomovirus*, *Curtovirus, Mastrevirus, and Topocuvirus*, depending on genome organization, host range and type of insect vectors [3]. The genome of cassava mosaic geminivirus (CMG) belonging to the genus *Begomovirus *consist of two components termed DNA A and DNA B each of ~ 2.7 - 3.0 kb [1,4]. The virus DNA A plus strand encodes the coat protein (CP/AV1) essential for viral transmission by whiteflies (*Bemisia tabaci*) [1,5]. There are three overlapping open reading frames (ORFs) on the complementary strand, of which the replication associated - protein (Rep/AC1) is absolutely required for the replication of both genomic components [6,7]. The replication enhancer protein (REn/AC3) is not essential for infection but enhances viral DNA accumulation [7]. The transcriptional activator protein (TrAP/AC2) is required for the transcription - activation of plus strand gene transcription, and is also involved in suppression of post - transcriptional gene silencing (PTGS) [8,9]. The functions of two other DNA A encoded proteins AV2 and AC4 remains unclear although possible roles in movement (AV2), pathogenicity and PTGS (AC4) have been demonstrated [9,10]. DNA B encodes the movement protein (BC1/MP) and a nuclear shuttle protein (BV1/NSP) required for cell - to - cell and long distance spread of virus in host plant [1]. Both DNAs contain a 200-250 bp region of high sequence homology known as the common region which is a part of a large intergenic region (IR) that contains the origin of replication [4].

Seven species of *Begomovirus *have been identified so far in association with cassava mosaic disease (CMD) in Africa: *African cassava mosaic virus *(ACMV), *East African cassava mosaic virus *(EACMV), *East African cassava mosaic Cameroon virus *(EACMCV), *East African cassava mosaic Kenya virus *(EACMKV), *East African cassava mosaic Malawi virus *(EACMMV), *East African cassava mosaic Zanzibar virus *(EACMZV) and *South African cassava mosaic virus *(SACMV) [11-14]. The distribution of the viruses has become more complex, since they invade new geographical regions and host plants [14,15]. This was attributed to their evolution which was more rapid than anticipated through mutational changes, recombination of double stranded (ds) DNA intermediates and re - assortment of gene components (pseudo - recombination) [12-17]. In fact, recombination played a role in the emergence of a new geminivirus that resulted in severe epidemics almost eliminating cassava (*Manihot esculenta*) in Uganda and Central Africa [12,18]. The symptom severity (due to synergism) was linked to the occurrence of *East African cassava mosaic virus *- Uganda (EACMV - UG) arising from recombination of EACMV and ACMV) [12]. Synergism refers to a situation where one virus affects a co - infecting virus by allowing its increased accumulation in the host plant by facilitating its replication, its movement to tissues that otherwise would not be invaded, resulting in more severe symptoms than caused by each single infection [19]. Synergism between EACMV and ACMV is due to a selective advantage conferred by each partner linked to post transcriptional gene silencing (PTGS). In plants, PTGS operates as an adaptive immune system targeted against viruses [20]. To counteract this defence system, viruses have developed suppressor proteins [21]. ACMV and EACMV - UG possess two PTGS suppressors AC4 and AC2 respectively, with differential roles that target different steps in RNA silencing in a temporal and spatial manner [8,10,21,22]. Therefore using more than one type of PTGS suppressor provides an advantage to viruses synergistically interacting in mixed infections, leading to more severe symptoms [9,10]. Furthermore, geminiviruses may be associated with small sub genomic DNA molecules termed as Defective (Def) DNAs, which are the result of partial deletion to approximately half the genome, even disrupting genes [23,24]. They may also result from sequence duplication, inversion or rearrangement of viral DNA, and recombination between DNA A and DNA B components [25].

Additionally, Cassava brown streak disease (CBSD), caused by *Ipomoviruses *(family *Potyviridae)*, has been reported to lead to severe yield losses in cassava plantations in Africa [26-28].

Both host plants under study, cassava and *Jatropha*, belong to the family *Euphorbiaceae*. *Jatropha *is a drought resistant shrub native in tropical America, but is now widely grown in many tropical and subtropical regions for biodiesel production [29,30]. Based on the genetic relationship of cassava and *Jatropha *and the detrimental impact of Begomoviruses in cassava, the question arose, whether *Jatropha *would be threatened by comparable epidemics, if planted on larger extensions, or in spatial neighbourhood. Therefore it was necessary to develop diagnostic approaches allowing reliable pathogen detection in *Jatropha*, which involved the amplification and sequencing of the entire DNA A molecules of 40 Kenyan isolates belonging to ACMV and EACMV - UG. This information enabled the design of novel primers to address different questions: a) primers amplifying longer sequences led to a phylogenetic tree of isolates, allowing some predictions on the evolutionary aspects of Begomoviruses in *Jatropha*; b) primers amplifying shorter sequences represent a reliable diagnostic tool, given that so far only limited serological tests are available.

## Results

### Symptomatology

*Jatropha *plants growing in the field showed symptoms ranging from reduced leaf size, malformation and severe dwarfing of 1 - 3 year old plants. Symptoms on *Jatropha *plants growing in the glasshouse were registered after 3 weeks as severe leaf yellowing coupled with browning of newly formed leaves, leaf malformation, reduced leaf size, mild to severe chlorotic specks and chlorosis in some plants. Drying (like burning) and rolling of leaves from tips was observed on 3 months old cuttings. In cassava the symptoms observed in plants the field grown and maintained in the glasshouse were similar: mosaic, severe reduction and distortion of leaves, and stunted growth of some plants.

### Virus detection by Enzyme-Linked Immunosorbent Assay (ELISA)

The *Jatropha *samples did not contain any of the three RNA viruses tested: *Cassava brown streak virus *(CBSV), *Cucumber mosaic virus *(CMV) and *Cassava common mosaic virus *(CsCMV) when analyzed by ELISA (see Additional file 1 Table S1). Also the cassava samples were negative for CMV and CsCMV. In fact, only three cassava samples from Busia district contained CBSV, as detected by ELISA (see Additional file 2 Table S2).

### Virus detection by Polymerase chain reaction (PCR)

All *Jatropha *and cassava samples were tested by PCR for the presence of geminiviruses. The primer pair JC3F and JC4R amplified longer sequences of DNA A which were used the construct a phylogenetic tree. Primer pair JC6F and JC2R amplified a shorter sequence of 380 bp from AC1, AC2 and AC3 and distinguished reliably positive from negative samples.

When symptomatic *Jatropha *samples from Kenya were tested with primer pair JC3F and JC4R, 69% were positive. The primers were able to further detect virus in 67% of asymptomatic samples. For symptomatic *Jatropha *samples collected from Ethiopia, 61% tested positive with the primers JC6F and JC2R (see Additional file 1 Table S1). With the same primers, 75% of symptomatic samples from Kenya tested positive while 20% of asymptomatic samples were detected positive.

For cassava, when symptomatic samples were amplified with primer pair JC3F and JC4R, 63% were positive while all asymptomatic samples tested negative. The primer pair JC6R and JC2R tested all (100%) symptomatic samples positive while only 6% of asymptomatic plants were positive (see Additional file 2 Table S2).

Samples testing positive with primer JC6R and JC2R yielded bands with different intensity on gel electrophoresis, which were classified as weak positive (+), moderate positive (++) and strong positive (+++) (see Additional file 1 Table S1 and Additional file 2 Table S2). Only 2 cassava samples were co - infected with CBSV and CMG (see Additional file 2 Table S2).

### Reverse transcription - polymerase chain reaction (RT-PCR)

All *Jatropha *samples tested negative for CMV and CBSV. The cassava samples were negative for CMV and only one sample tested positive for CBSV.

### Sequences and phylogenetic analysis of DNA A

Complete nucleotide sequences of forty DNA A components typical of Begomoviruses in the Kenyan samples were determined, of which 34 sequences were from *Jatropha *and 6 from cassava (see Additional file 3 Table S3).

**Figure 1:**
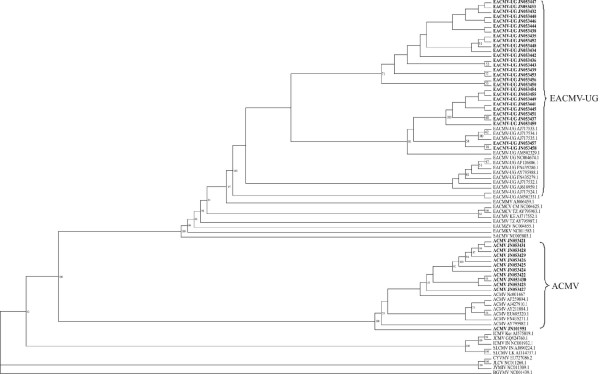
**Phylogenetic alignment of nucleotide sequences of cassava mosaic geminivirus isolates obtained from *Jatropha *and cassava in the study and other related Begomoviruses from the Genbank**. Viruses obtained from the study have been written in bold. Geminivirus type group species BGYMV was used as an out group. Abbreviations and Genbank Accession numbers are given in Table 1.

Figure 1 shows a phylogenetic comparison of the complete DNA A sequences of the Begomoviruses isolates obtained from *Jatropha *and cassava in this study and other Begomoviruses associated with the two host plants publicly available in the Genbank (Table 1) [12-14,16,17,31-40]. Phylogenetic analyses clearly indicated that they belong to CMG involved in CMD as they had close identities with sequences already deposited in public databases. All viruses characterised in this study could be grouped with two previously identified Begomoviruses found in cassava in Western Kenya, namely EACMV - UG and ACMV, but not with the species EACMKV [GenBank: NC011583.1], EACMZV [GenBank: NC004655.1] and strain EACMV - KE [GenBank: AJ717552.1], which were found in Kenya previously [14]. The viruses also did not group with EACMV - TZ [GenBank: AY795987.1], EACMCV - TZ [GenBank: AY795983.1], EACMCV - CM [GenBank: NC004625.1], EACMMV [GenBank: AJ006459.1], SACMV [GenBank: NC003803.1], ICMV - IN [GenBank: NC001932.1], ICMV - ker [GenBank: AJ575819.1], SLCMV - IN [GenBank: AJ890224.1] and SLCMV - LK [GenBank: AJ314737.1] found elsewhere in Africa and Asia [12,13,36-38]. No close relationship was shown between the viruses and *Jatropha *begomoviruses from Asia and South America; JCMV [GenBank: GQ924760.1], CYVMV [GenBank: EU727086.2], JYMIV [GenBank: NC011309.1] and JLCV [GenBank: NC011268.1].

**Table 1:** Geminiviruses used for comparisons, assigned abbreviations and their genomic sequence accession numbers

Virus	Abbreviation	Genbank accession number	Reference
*African cassava mosaic virus*	ACMV	FN435271.1	[31]
	ACMV	EU685320.1	[32]
	ACMV	AF259894.1	[17]
	ACMV	AJ427910.1	[unpublished; Briddon]
	ACMV	AY211884.1	[16]
	ACMV	NC001467.1	[33]
	ACMV	AY795982.1	[13]
*Bean golden yellow mosaic virus*	BGYMV	NC001439.1	[34]
*Croton yellow vein mosaic virus*	CYVMV	EU727086.2	[unpublished; Raj et al.]
*East African cassava mosaic Cameroon virus *- Cameroon	EACMCV - CM	NC004625.1	[16]
*East African cassava mosaic Cameroon virus *- Tanzania	EACMCV - TZ	AY795983.1	[13]
*East African cassava mosaic virus *- Kenya	EACMV - KE	AJ717552.1	[14]
*East African cassava mosaic virus *- Tanzania	EACMV - TZ	AY795987.1	[13]
*East African cassava mosaic virus *- Uganda	EACMV - UG	AJ618959.1	[35]
	EACMV - UG	FN435279.1	[31]
	EACMV - UG	NC004674.1	[17]
	EACMV - UG	AJ717524.1	[14]
	EACMV - UG	AJ717532.1	[14]
	EACMV - UG	AJ717533.1	[14]
	EACMV - UG	AJ717534.1	[14]
	EACMV - UG	AJ717535.1	[14]
	EACMV - UG	AM502329.1	[35]
	EACMV - UG	AM502331.1	[35]
	EACMV - UG	AF126806.1	[17]
	EACMV - UG	FN435280.1	[31]
	EACMV - UG	AY795988.1	[13]
*East African cassava mosaic Kenya virus *	EACMKV	NC011583.1	[14]
*East African cassava mosaic Malawi virus *	EACMMV	AJ006459.1	[12]
*East African cassava mosaic Zanzibar virus *	EACMZV	NC004655.1	[36]
*Indian cassava mosaic virus *- India	ICMV - IN	NC001932.1	[37]
*Indian cassava mosaic virus *- Kerela	ICMV - Ker	AJ575819.1	[38]
*Jatropha curcas mosaic virus*	JCMV	GQ924760.1	[30]
*Jatropha leaf curl virus*	JLCV	NC011268.1	[unpublished; Pal&Mukherjee]
*Jatropha yellow mosaic India virus *	JYMIV	NC011309.1	[unpublished; Raj et al.]
*South African cassava mosaic virus*	SACMV	NC003803.1	[39]
*Sri Lankan cassava mosaic virus *- India	SLCMV - IN	AJ890224.1	[40]
*Sri Lankan cassava mosaic virus *- Sri Lanka	SLCMV - LK	AJ314737.1	[38]

The first group of viruses comprises ACMV, showing nucleotide (nt) identity from 95% (isolate JN053426) to 97.3% (isolates JN053431 and JN053430) with the ACMV reference sequence [GenBank: NC001467.1] (see Additional file 3 Table S3). The second group are closely related to, but distinct from the strains EACMV - KE and EACMV - TZ. The sequences showed approximately 90.7% nt identity (JN053450) to 92.3% nt identity (JN053440 and JN053444) with the EACMV - KE [GenBank: AJ717552.1] and only 90% nt identity (JN053451, JN053441, JN053452 and JN053453) to 90.5% nt identity (JN053433, JN053440, JN053442, JN053444 and JN053446) with EACMV - TZ [GenBank: A1795987.1]. To indicate that they are clearly isolates of the strain EACMV - UG, they had high nt identity ranging from approximately 94.1% (isolate JN053439) to 98.7% (isolates JN053440, JN053442 and JN05344) with the EACMV - UG [GenBank: NC004674.1] reference sequence (see Additional file 3 Table S3).

The viruses infecting *Jatropha *in Western Kenya occur on overlapping territories, since ACMV and EACMV - UG were both found in all the districts analysed (see Additional file 3 Table S3). EACMV - UG occurred with a higher prevalence than ACMV. Generally speaking, out of 34 viral sequences found in *Jatropha *24 (71%) were EACMV - UG, while 10 (27%) were ACMV. In Busia, a district neighbouring Uganda, EACMV - UG was most prevalent with 15/24 (62.5%) compared to 7/24 (29%) and only 2/24 (8%) from Kakamega and Siaya respectively. ACMV was found more frequently with 6/10 (60%) on samples from Kakamega district, compared to 2/10 (20%) from Siaya and Busia respectively.

### ACMV Def DNA A

**Figure 2:**
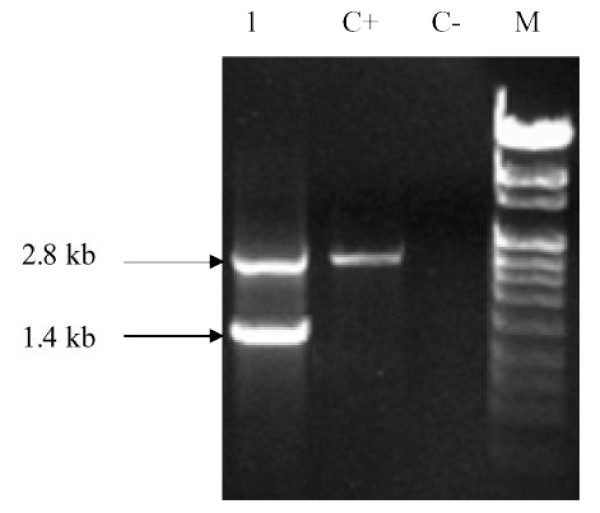
**PCR amplification of defective DNA A of ACMV from *Jatropha***. PCR amplification was performed using primer JC3F and JC4R on DNA extracted from field - grown *Jatropha *plants. Lane 1 shows the defective DNA A, lane C+ and C- positive and negative controls. Lane M = marker VIII (Roche Applied Science).

**Figure 3:**
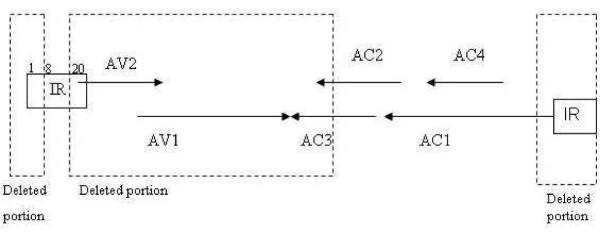
**Schematic genome organization of subgenomic Def K1J5**. Predicted ORFs in both directions: AC1 - 4 are complementary sense strands; AV1 and AV2 are virion sense strands. The deleted part of the genome based on the known genome of a full length DNA A component of ACMV [GenBank NC001467] is shown (dashed). Nucleotide positions 1, 8 and 20 are indicated.

A Def DNA A was present in a *Jatropha *leaf samples collected from Western Kenya. PCR analyses with primer JC3F and JC4R of sample K1J5 amplified the expected 2.8 kb of a near full length DNA A component of the Begomovirus sequence and an additional shorter fragment (Figure 2). Sequencing of the smaller fragment revealed a size of 1420 bp, which was named Def K1J5. This Def (Genbank JN101951) showed 96.6% nt identity with the ACMV reference sequence [GenBank NC001467.1] and a low nt similarity (69.7%) with the EACMV - UG reference sequence [Genbank NC004674.1] (see Additional file 3 Table S3). The complete sequences of DNA A components of ACMV reference sequence [GenBank NC001467.1] was used for size comparison with the ORFs of Def K1J5. On the virion sense strand, AV1 and AV2 were entirely missing. In the complementary sense strand, AC4 was the only intact gene at 422 bp while AC1 was 1070 bp long. Two ORFs, AC2 and AC3, which are found in DNA A of CMGs, had a size of 326 bp and 178 bp respectively (Figure 3). The IR contained the first 11 bp of the replication site of geminiviruses.

## Discussion

Cultivated cassava is believed to be the principle reservoir for CMD associated begomoviruses because of its perennial growth and scale of production. However, alternative hosts have been identified, including *Manihot glaziovii *Müll, a wild relative of cassava native to Brazil [35], *Senna occidentalis, Leucana leucocephala, Combretum confertum, Centrosema pubescens *and *Pueraria javanica *[31,32]. A strain of *Cassava latent geminivirus *(CLV - V) was previously isolated from naturally infected *Jatropha multifida *growing in the Coastal districts of Kenya [41]. First reports on geminivirus infections on *J. curcas *indicated the occurrence of *Jatropha curcas virus *closely related to *Cassava mosaic virus *in India, reaching a disease incidence from 25 to 47% [30,42,43]. Phylogenetic analyses of the virus genome showed that *Jatropha curcas *mosaic disease (JCMD) is caused by *Jatropha curcas virus*, a new strain of *Indian cassava mosaic virus *(ICMV) [30]. *Jatropha *is further host of CMV (family *Bromoviridae Cucumovirus*) [44]. In this study, we report for the first time the detection of *Begomovirus*: ACMV and EACMV - UG in *Jatropha*. Furthermore, a defective molecule derived from DNA A of the bipartite *Begomovirus *ACMV was detected in *J. curcas*. Also the presence of a co **- **infection with CMG and CBSV was found in cassava plants.

The phylogenetic tree of the complete DNA A sequences indicates that the ACMV and EACMV - UG isolates were closely related to those isolated previously in Western Kenya [14,33]. From an evolutionary perspective, it is an indication that the geminiviruses infecting *Jatropha *from the sampled areas are as a result of spread of viruses from an inoculum source occurring where the plants are growing. This is further supported by the idea that the EACMKV, EACMV - KE and EACMZV previously identified [14] from Eastern and Coastal parts of Kenya were not found to be present in *Jatropha*, since the viruses have a distinct geographical distribution [14]. Geminivirus dissemination occurs through cuttings or whiteflies. The viruses identified in this study were not closely related with those infecting cassava and *Jatropha *in other parts of Africa, Asia and South America suggests that there has been no movement of infected *Jatropha *cuttings and viruliferous whiteflies from other areas to Western Kenya. In fact, recombination results in severe epidemiological consequences such as the emergence of isolates with increased virulence capable of overcoming host resistance or with a host range wider than the original one [45]. Recombination and synergism that have long occurred in cassava [12-18] could have led to the current spread of the virus in the field to infect *Jatropha *plants. The recombinant EACMV - UG was the most prevalent strain virus found whilst the other strains of EACMV were not identified. In line with this are previous claims, that EACMV in Western Kenya has been largely displaced by EACMV - UG, which is considered a more virulent strain [14]. In the current study the presence of EACMV - UG and ACMV on different *Jatropha *plants in the same field indicates the opportunity for mixed infections. For example plants K4J1 (EACMV - UG isolate JN053453) and K4J2 (ACMV isolate JN053425) (Figure 1) stand close to each other in the same field, hence offering good opportunities for more recombination to occur. EACMV - UG and ACMV are associated with severe synergistic epidemics on cassava that swept through Uganda and continues to affect surrounding countries including Kenya [14,12,18,46]. Specifically the two viruses have differentially acting suppressors of PTGS overcoming the hosts defence mechanisms [8-10,20-22]. ACMV (recovery - type) has a strong AC4 suppressor and EACMV - UG (non - recovery - type) has a strong AC2 suppressor causing unusually severe symptoms. As a result, ACMV will leave only EACMV - UG to be spread and become the predominant virus in the area [9,21,47]. In the absence of a synergistic interaction, only one virus in a co - infected plant will become predominant and persist [9,10,14]. Synergism may lead to a 10 - 50 fold increase in viral DNA accumulation which substantially increases the potential for a higher efficiency of vector transmission to even infect non cassava host plants [18,31] This might further explain, why the EACMV - UG appears as predominant virus in *Jatropha*. Co - infection of CMG and CBSV threatens cassava production in Busia distict of Kenya. Recent studies have shown how evolution is shaping the populations of CBSV and *Uganda cassava brown streak virus *(UCBSV) in cassava causing significant problems [26-28]. Mixed infection results in increase in the titer of one or both viruses and elicits disease symptoms that are more sever than the sum of those induced in single infection [10,19].

In addition to genomic components, smaller sized Def DNA often occurs naturally in geminivirus infected plants [25]. The plant, from which the defective DNA molecule was isolated in the current study, did not display particular symptoms differing from the neighbouring plants growing in the same field, meaning that it could not have been picked up on purpose due to a previous selective decision. The Def DNA molecule found in the plant K1J5 had lost the entire AV1 and AV2 genes and large portions of other genes. Sub genomic Def DNA molecules associated with a number of Begomovirus seem to be fairly uniform in structure and retain their IR and a large portion of AC1 [23], as observed also in this study. These deletions might affect the replication of the molecule and it might depend entirely on its helper virus for replication. Geminivirus Def DNA invariably rely on the respective viruses for replication as observed for *Tomato leaf curl virus *that lacked an ORF required for replication and encapsidation and were not expected to be capable of autonomous replication, however they were replicating in the presence of the viral DNA [48]. In cassava, Def DNA has been previously reported occurring in DNA A of EACMV and DNA B of ACMV, and were found to be associated with a delay in symptom development and amelioration [23,24]. However, no naturally occurring Def DNA A of ACMV has been found previously in *Jatropha curcas *and we report it for the first time. The role of this small Def DNA molecule in the biology of ACMV in *Jatropha *in nature is still unclear.

## Conclusion

We have shown for the first time cassava Begomoviruses and their associated sub - genomic Def DNA molecules to be naturally occurring in field growing *Jatropha *plants. The occurrence of the Begomoviruses further poses a challenge in the elimination strategy of CMG in field grown cassava as a result of increase in inoculum from different hosts, and calls for an elimination strategy of the viruses in *J. curcas *in order to save the crop which is an important biofuel and pharmaceutical crop. Molecular detection techniques showed the presence of geminivirus even in asymptomatic plants. The new natural host (*J. curcas*) of the two viruses opens new avenues for further recombination of the viruses to occur which indeed becomes a threat both to cassava an important food crop to Sub Saharan countries and *Jatropha*. There is a possibility of *Cassava mosaic virus *in *Jatropha *being more widespread than anticipated, since we have detected it also in *Jatropha *samples from Ethiopia. This has led to hypothesize that other neighboring countries growing *Jatropha *could be facing similar challenges with this plant. The primer pair JC6F and JC2R amplifying a sequence of 380 bp allows the detection of Begomoviruses in symptomatic and asymptomatic cassava and *Jatropha *plants and can therefore be recommended for a large scale screening of field samples.

## Methods

### Sample collection

**Figure 4:**
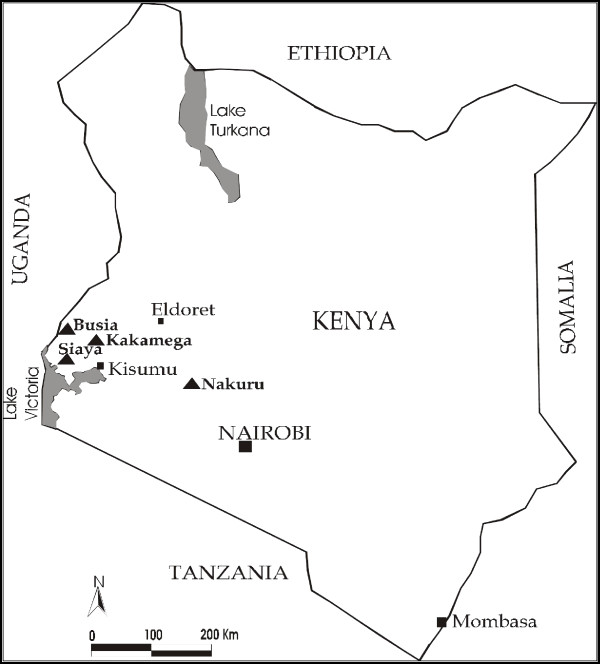
**Map of Kenya showing the sites of cassava and *Jatropha *plant material sampling (black triangles)**. A survey was conducted in Western Kenya and Rift valley where the two plants are being grown together.

A total of 127 *Jatropha *samples from Ethiopia and Kenya and 124 cassava samples from Kenya were used in this study. The Kenyan samples were collected during a survey conducted in September 2009 and November 2010 covering four districts: Kakamega, Siaya, Busia (Western region) and Nakuru (Rift valley region) growing *Jatropha *and cassava together (Figure 4). Ten plants of *Jatropha *and cassava showing typical virus symptoms and ten symptomless plants were sampled from five fields in each district. Young leaves were picked from the plants and placed in sample collection tubes over silica gel for further detection of viruses. Two cuttings of approximately 30 cm long were also taken from 5 symptomatic and 5 asymptomatic plants and planted in glasshouse for future use.

### Enzyme-Linked Immunosorbent Assay

Double Antibody Sandwich ELISA (DAS - ELISA) was performed on all *Jatropha *and cassava plant accessions to detect the presence of RNA viruses such as: CMV and CsCMV using commercially available kits (DSMZ GmbH, Germany and AC Diagnostics). Triple - Antibody Sandwich - ELISA (TAS - ELISA) was performed to determine the presence of CBSV (DSMZ GmbH, Germany). An ELISA sample was taken as positive, when its OD value was at least three times higher than the negative control. All determinations were run in duplicate.

### DNA extraction and rolling cycle amplification (RCA)

Total genomic DNA was extracted from leaves using the DNeasy plant Mini Kit (QIAGEN, Hilden, Germany) according to the supplier's instructions. RCA was performed using the TempliPhiTM Kit (Amersham Biosciences) according to the supplier's instructions.

### Polymerase chain reaction

Six different primers were designed (Table 2) based on multiple alignments of full length DNA A sequence of geminivirus from *Jatropha *and cassava available in the NCBI Genbank to amplify the variable regions to yield longer (2800 bp) and shorter sequences (380 - 1085 bp) (Table 3). PCR was conducted in a total volume of 25 μl using 2.5 μl 10× PCR buffer (QIAGEN), 1 μl MgCl_2 _(25 mM), 0.5 μl of each primer (10 pmol), 0.5 μl dNTP, 0.15 μl HotStarTaq Polymerase (QIAGEN HotStar Plus TM PCR), 1 μl of total genomic DNA or RCA (1:30) product. Total genomic DNA was used with all the primer sets that amplify shorter sequences while the RCA was used for primer sets that amplify longer sequences (Table 3). For RCA to amplifying longer sequences, the PCR cycling conditions consisted of an initial denaturation step of 95°C for 5 min followed by 35 cycles of 1 min at 94°C, 1 min annealing temperature (Table 3) and 2 min at 72°C. A final step of 10 min at 72°C ended the cycle. For primers pairs amplifying shorter sequences, the difference in PCR conditions was in 30 cycles of 40 s at 94°C and 40 sec annealing temperature (Table 3). The PCR products were analyzed by electrophoresis in a 1% agarose gel. A subsequent purification of full length PCR products was done using QIAquick PCR purification kit (QIAGEN).

**Table 2:** List of the oligonucleotides used in this study

Primer designation*	Primer sequence (5' to 3')
JC1F	GGAAGATAGTGGGAATGCCNCCTTTAATTTGAA
JC2R	AARGAATTCATGGGGGCCCARAGRGACTGGC
JC3F	RTCGACGTCATCAATGACGTTGTACCAKGCG
JC4R	GTHGAYCCSCACTAYCTMAARCACTTCAARG
JC5R	GGCCATCCGGTAATATTAWWCGGATGG
JC6F	CCATTCATTGCTTGAGGAGCAGTG

*Primer designation: F denotes forward R means reverse.

Primer sequence: R represents A or G; K represents G or T; H represents A, C or T; Y represents C or T; S represents G or C; M represents A or C; W represents A or T.

**Table 3:** Primer combinations and annealing temperatures used to detect geminiviruses in *Jatropha curcas *and *Manihot esculenta *in PCR and RCA

Forward primer	Reverse primer	Length	Annealing temperature	Part of genome amplified by PCR and RCA
JC1F	JC5R	1085 bp	60°C	PCR : amplifies part of AC1 and entire AC4
JC3F	JC5R	971 bp	63°C	PCR : amplifies part of AC1 and entire AC4
JC3F	JC4R	2800 bp	64°C	RCA : amplifies the entire DNA A
JC3F	JC2R	2800 bp	64°C	RCA : amplifies the entire DNA A
JC6F	JC4R	410 bp	55°C	PCR : amplifies part of AC2 and AC3
JC6F	JC2R	380 bp	55°C	PCR : amplifies part of AC1, AC2 and AC3

### Extraction of RNA and RT-PCR

RT-PCR was performed to detect CBSV and CMV in *Jatropha *and cassava. Total RNA was extracted from 100 mg of *Jatropha *and cassava leaves using Spectrum ™ plant total RNA kit (SIGMA - ALDRICH) according to the supplier's instructions. cDNA was synthesized from 3 μg of genomic RNA using SuperScript II ™reverse transcriptase primed with oligo(dT)_12-18 _(Invitrogen). The CBSV specific primers CBSV 10F: 5'ATCAGAATAGTGACTGCTGG 3' and CBSV 11R: 5' CCACATTATTATCGTCACCAGG 3' [49] amplifying 230 bp were used for PCR amplification of the cDNA template. The reaction mix and PCR cycling conditions were as performed previous [49]. For CMV detection, Cucumoviruses universal primers CPTALL - 3: 5' GACTGACCATTTTAGCCG 3' and CPTALL - 5: 5' YASYTTTDRGGTTCAATTCC 3' [50] amplifying 940 bp were used for PCR with the reaction mixture and cycling conditions as described previously [50].

### Sequence analyses

Multiple sequence alignments of geminivirus full length DNA A sequences was carried out using the Clustal program (MegAlign, DNAStar). A phylogenetic tree was constructed from multiple alignments by performing a heuristic search. Multiple alignments were analyzed by maximum parsimony with full-length DNA A using Phylogenetic Analysis Using Parsimony (PAUP) and a bootstrap analysis with 1000 replicates was performed.

## Competing interests

The authors declare that they have no competing interests.

## Authors' contributions

Design and conception of the study, execution of the experiments (RCR, FM, ML), sequence analysis, alignment and phylogeny (RCR, AC, FM, ML). All authors read and approved the final manuscript.

## Supplementary Material

Additional file 1 **Table S1: The summary of *Jatropha curcas *plants tested for CMG, CBSV, CsCMV and CMV**. Click here for file

Additional file 2 **Table S2: The summary of *Manihot esculenta *plants tested for CMG, CBSV, CsCMV and CMV**. Click here for file

Additional file 3 **Table S3: Nucleotide sequence identities of DNA - A full length of *Jatropha *and Cassava geminiviruses from Kenya and other geminiviruses available in Genbank**. Click here for file
