# Supplementary material for: Biotechnological approaches to determine the impact of viruses in the energy crop plant Jatropha curcas
Source: Virol J. 2011 Aug 3;8:386. doi: 10.1186/1743-422X-8-386 (PMC3163225; doi:10.1186/1743-422X-8-386)
Supplement: Additional file 1 — Table S1: The summary of Jatropha curcas plants tested for CMG, CBSV, CsCMV and CMV. [file 1743-422X-8-386-S1.PDF]

Table S1 *Jatropha curcas* plants tested for the viruses: Cassava mosaic geminivirus (CMG) by PCR with primer JC6F and JC2R, *Cassava brown streak virus* (CBSV), *Cassava common mosaic virus* (CsCMV), *Cucumber mosaic virus* (CMV) using ELISA. - negative samples, + positive samples; weak positive (+).

Origin		Plants		PCR	ELISA		
Country	District	Symptomatic	Asymptomatic	CMG	CBSV	CsCMV	CMV
Kenya	Busia	BxJ1		-	-	-	-
		BxJ4		+	-	-	-
		BxJ5		+	-	-	-
			B1J11	-	-	-	-
			B1J14	-	-	-	-
			BxJ15	+	-	-	-
		B2J2		+	-	-	-
		B2J3		-	-	-	-
		B2J5		+	-	-	-
			B2J13	+	-	-	-
			B2J16	-	-	-	-
		B3J1		+	-	-	-
		B3J2		+	-	-	-
		B3J3		+	-	-	-
			B3J4	-	-	-	-
			B3J13	+	-	-	-
			B3J14	-	-	-	-
		B4J2		-	-	-	-
		B4J3		+	-	-	-
		B4J5		+	-	-	-
			B4J15	+	-	-	-
		B5J1		+	-	-	-
		B5J2		+	-	-	-
		B5J3		+	-	-	-
			B5J15	+	-	-	-
	Kakamega	K1J1		+	-	-	-

	K1J3		+	-	-	-
	K1J5		+	-	-	-
		K1J12	-	-	-	-
		K1J14	-	-	-	-
		K1J16	+	-	-	-
	K2J1		-	-	-	-
	K2J3		+	-	-	-
	K2J4		+	-	-	-
		K2J8	-	-	-	-
		K2J16	-	-	-	-
	K3J1		-	-	-	-
	K3J2		-	-	-	-
	K3J3		-	-	-	-
	K3J4		+	-	-	-
		K3J5	-	-	-	-
		K3J7	-	-	-	-
		K3J11	-	-	-	-
		K3J12	-	-	-	-
		K3J15	-	-	-	-
		K3J16	+	-	-	-
		K3J17	-	-	-	-
	K4J1		+	-	-	-
	K4J2		+	-	-	-
	K4J3		+	-	-	-
		K4J15	-	-	-	-
		K4J16	-	-	-	-
		K4J20	+	-	-	-
		K5J2	-	-	-	-
	K5J3		-	-	-	-
	K5J5		+	-	-	-
	K5J6		+	-	-	-
		K5J11	-	-	-	-
		K5J18	+	-	-	-
Siaya	S1J1		-	-	-	-

	S1J2		-	-	-	-
	S1J3		-	-	-	-
	S1J4		-	-	-	-
		S1J6	-	-	-	-
		S1J12	+	-	-	-
		S1J13	-	-	-	-
		S1J15	-	-	-	-
		S1J16	-	-	-	-
	S4J1		+	-	-	-
	S4J2		-	-	-	-
	S4J3		-	-	-	-
		S4J11	-	-	-	-
		S5J1	+	-	-	-
		S5J3	+	-	-	-
Nakuru	N1J1		-	-	-	-
	N1J2		-	-	-	-
	N1J3		-	-	-	-
	N1J5		-	-	-	-
		N1J6	-	-	-	-
		N1J8	-	-	-	-
		N1J10	-	-	-	-
		N2J3	+	-	-	-
		N2J6	-	-	-	-
		N2J7	-	-	-	-
		N2J8	-	-	-	-
		N2J9	-	-	-	-
		N2J10	-	-	-	-
		N3J9	-	-	-	-
		N3J10	-	-	-	-
	N4J1		-	-	-	-
	N4J2		-	-	-	-
		N4J6	-	-	-	-
		N4J10	-	-	-	-
	N5J1		-	-	-	-

	N5J2		-	-	-	-
	N5J5		-	-	-	-
		N5J8	-	-	-	-
Ethiopia	PIC 440		+	-	-	-
	PIC 610		-	-	-	-
	PIC 449		+	-	-	-
	PIC 423		+	-	-	-
	PIC 547		-	-	-	-
	PIC 555		-	-	-	-
	PIC 547		+	-	-	-
	PIC 381		+	-	-	-
	PIC 611		-	-	-	-
	PIC 448		-	-	-	-
	PIC 481		+	-	-	-
	Sample 42		+	-	-	-
	Sample 43		+	-	-	-

.
