# Supplementary material for: Biotechnological approaches to determine the impact of viruses in the energy crop plant Jatropha curcas
Source: Virol J. 2011 Aug 3;8:386. doi: 10.1186/1743-422X-8-386 (PMC3163225; doi:10.1186/1743-422X-8-386)
Supplement: Additional file 2 — Table S2: The summary of Manihot esculenta plants tested for CMG, CBSV, CsCMV and CMV. [file 1743-422X-8-386-S2.PDF]

Table S2 ***Manihot esculenta*** **plants tested for Cassava mosaic geminivirus (CMG) by PCR with primer JC6F and JC2R, as well as** ***Cassava brown streak virus*** **(CBSV),** ***Cassava common mosaic virus*** **(CsCMV) and** ***Cucumber mosaic virus*** **(CMV) by ELISA. - negative samples, + positive samples; weak positive (+), moderate positive (++) and strong positive (+++). Plants in Bold are co – infected with CMG and CBSV.**

Origin	Plants		PCR	ELISA		
District	Symptomatic	Asymptomatic	CMG	CBSV	CsCMV	CMV
Kakamega		K1C5	-	-	-	-
	K1C8		+	-	-	-
	K2C1		+	-	-	-
		K2C2	-	-	-	-
	K2C3		+	-	-	-
	K2C4		+	-	-	-
	K2C5		+++	-	-	-
		K2C6	-	-	-	-
	K2C15		+++	-	-	-
		K3C1	-	-	-	-
		K3C2	-	-	-	-
		K3C3	-	-	-	-
	K3C4		+++	-	-	-
		K3C5	+	-	-	-
		K3C13	-	-	-	-
		K3C15	-	-	-	-
	K3C17		+++	-	-	-
	K4C6		+++	-	-	-
	K4C7		+++	-	-	-
		K4C15	-	-	-	-
	K5C6		+++	-	-	-
	K5C7		+	-	-	-

		K5C13	-	-	-	-
		K5C17	-	-	-	-
Busia		B1C1	-	-	-	-
		B1C2	-	-	-	-
	B1C3		+	-	-	-
	B1C5		+	-	-	-
		B1C6	-	-	-	-
		B1C11	-	-	-	-
		B1C12	-	-	-	-
		B1C14	-	-	-	-
	B1C15		++	-	-	-
	B2C5		+++	-	-	-
	B2C12		+	-	-	-
		B2C15	-	-	-	-
		B2C16	-	-	-	-
	B2C19		+	-	-	-
	B3C2		+++	-	-	-
	**B3C3**		+++	+	-	-
	B3C4		+++	-	-	-
	B3C5		+	-	-	-
	B3C11		+	-	-	-
		B3C12	-	-	-	-
		B3C13	+	-	-	-
		B2C14	-	-	-	-
		B3C15	-	-	-	-
		B3C17	-	-	-	-
		B4C2	-	-	-	-
		B4C3	+	-	-	-
		B4C4	-	-	-	-
	B4C5		+++	-	-	-
		B4C11	-	-	-	-

		B4C12	-	+	-	-
		B4C16	-	-	-	-
	**B5C1**		++	+	-	-
		B5C11	-	-	-	-
		B5C12	-	-	-	-
	B5C14		+	-	-	-
Siaya	S1C3		++	-	-	-
	S1C4		++	-	-	-
		S1C11	+	-	-	-
		S1C13	-	-	-	-
		S2C1	-	-	-	-
		S2C2	-	-	-	-
		S2C4	-	-	-	-
	S2C6		++	-	-	-
		S2C12	-	-	-	-
		S2C13	-	-	-	-
		S2C14	-	-	-	-
	S3C5		++	-	-	-
		S3C12	-	-	-	-
		S3C14	-	-	-	-
	S4C3		+	-	-	-
	S4C4		+	-	-	-
	S4C6		++	-	-	-
		S4C11	-	-	-	-
		S4C12	-	-	-	-
		S4C14	-	-	-	-
	S4C15		++	-	-	-
	S5C2		++	-	-	-
	S5C4		++	-	-	-
	S5C6		++	-	-	-
	S5C7		++	-	-	-

	S5C12	-	-	-	-
	S5C13	+	-	-	-
S5C14		+	-	-	-
	S5C18	-	-	-	-
	S5C19	-	-	-	-
