# Supplementary material for: Biotechnological approaches to determine the impact of viruses in the energy crop plant Jatropha curcas
Source: Virol J. 2011 Aug 3;8:386. doi: 10.1186/1743-422X-8-386 (PMC3163225; doi:10.1186/1743-422X-8-386)
Supplement: Additional file 3 — Table S3: Nucleotide sequence identities of DNA - A full length of Jatropha and Cassava geminiviruses from Kenya and other geminiviruses available in Genbank. [file 1743-422X-8-386-S3.PDF]

Table S3 Nucleotide sequence identities (percentages) of the DNA - A full length of *Jatropha* and cassava geminiviruses from Kenya and other geminivirus publicly available in the Genbank. Values above 90% are in bold.

District	Nearest collection village/Location	Genbank accession numbers of Virus isolate*	ACMV	EACMV – UG	EACMV – KE	EACMV – TZ	EACMKV	EACMZV	SACMV	EACMMV	EACMCV – CM	ICMV – Ker	JCMV	JYMIV	JLCV
Busia	Bujuan´ga	JN053436	75.3	**98.4**	**91.9**	**90.1**	83.6	83.2	79.2	87	84	69.7	69.6	66.4	63.7
	Bujuan´ga	JN053443	75.4	**98.4**	**91.9**	**90.1**	83.6	83.3	79.2	87.1	84.1	69.7	69.7	66.3	63.4
	Bujuan´ga	JN053451	75.3	**98**	**91.7**	**90**	84	83.5	79.6	87.1	83.8	69.6	69.5	66.8	63.9
	Khayinga	JN053447	75.6	**98.6**	**92.2**	**90.4**	83.7	83.4	79.1	87.1	84.3	69.8	69.9	66.4	63.6
	Khayinga	JN053432	75.6	**98.5**	**92.1**	**90.4**	83.7	83.3	79.1	87	84.2	69.6	69.7	66.3	63.6
	Sibinga	JN053457*	77.3	**95.4**	89.3	87.5	82.5	82.5	78.1	84.3	82.8	69.8	69.9	66.4	63.7
	Khayinga	JN053439	77	**94.1**	88.5	86.6	82.6	82.2	78.5	84.5	81.2	68.7	68.9	65.6	62.5
	Khayinga	JN053433	75.6	**98.6**	**92.2**	**90.5**	83.8	83.4	79.2	87.2	84.2	69.7	69.8	66.4	63.7
	Khayinga	JN053440	75.6	**98.7**	**92.3**	**90.5**	83.9	83.4	79.3	87.2	84.3	69.2	69.9	66.4	63.6
	Mulakha ganja	JN053459*	75.3	**98.4**	**92.2**	**90.3**	84.1	83.7	79.6	87	84.2	69.9	69.7	66.8	64
	Khayinga	JN053448	75.5	**97.7**	**91.7**	89.9	83.8	83.2	79.3	87.1	84.1	69.8	69.3	66.3	63.6
	Khayinga	JN053434	75.5	**98.6**	**92.2**	**90.4**	83.8	83.4	79.3	87.2	84.2	69.9	70	66.4	63.7
	Nambugu	JN053441	75.1	**98.1**	**91.9**	**90**	83.9	83.5	79.5	86.9	83.8	69.7	69.6	66.7	63.8
	Nambugu	JN053449	75.3	**98.3**	**92**	**90.2**	84.1	83.6	79.6	87.1	84	69.8	69.7	66.8	63.6
	Nambugu	JN053435	75.4	**97.3**	**91.4**	89.7	83.7	83.1	79.3	87.2	83.8	69	69.2	65.9	63.5
	Funyula	JN053422	**96.9**	75	71.3	70.8	74.3	73.1	74.3	72.8	68.6	70.6	70.9	68.9	64.2
	Funyula	JN053423	**97.1**	75.3	71.7	70	74.9	73.5	74.7	73.2	68.9	71	71.2	68.9	64.3
	Funyula	JN053442	75.6	**98.7**	**92.2**	**90.5**	83.8	83.4	79.3	87.1	84.1	69.7	69.8	66.4	63.7
	Funyula	JN053450	75.7	**96.9**	**90.7**	88.9	83.6	82.9	79.3	85.9	82.8	69.6	69.6	66.2	63.6
Kakamega	Musumba	JN053421	**96.2**	74.3	71	70.6	74.1	72.7	73.6	72.2	68.4	70.3	70.7	74.7	63.7
	Musumba	JN053444	75.7	**98.7**	**92.3**	**90.5**	83.8	83.5	79.3	87.2	84.3	69.8	69.9	66.4	63.7
	Musumba	JN101951	**96.6**	69.7	69.5	69.6	76	74.4	75.9	70.7	67.4	72.5	73.1	69.9	66.1
	Musumba	JN053424	**97**	75.2	71.5	70.9	74.7	73.2	74.3	72.8	69	70.8	71.2	68.3	64.1
	Matungu	JN053452	76.1	**98**	**91.8**	**90**	83.9	83.5	79.3	87.2	84.2	69.7	69.9	66.5	63.7
	Matungu	JN053437	75.3	**98.4**	**92.1**	**90.3**	84.1	83.8	79.7	87.2	84.1	69.8	69.7	66.9	64.1
	Lung´anyiro	JN053445	75.3	**98.3**	**92**	**90.2**	84.1	83.7	79.6	87.1	84.1	69.8	69.7	66.8	63.9
	Lung´anyiro	JN053427	**95.3**	74.1	70.2	69.5	73.4	72	73.3	71.6	67.3	69.8	70.2	67.4	62.6
	Matungu	JN053453	75.6	**98.1**	**91.7**	**90**	83.8	83.8	79.1	86.7	83.8	69.6	69.8	66.1	63.7

	Musamba	JN053430*	**97.3**	75.1	71.4	70.7	74.4	73.1	74.6	73	68.7	70.8	71	69	64.4
	Matungu	JN053425	**97.4**	75.2	71.5	71.1	74.7	73.4	74.6	72.8	68.9	70.8	71.2	68.4	64
	Matungu	JN053438	75.6	**98.6**	**92.2**	**90.4**	83.7	83.4	79.1	87.1	84.2	69.7	69.6	66.3	63.6
	Matungu	JN053428	**97.2**	75.1	71.7	71.2	74.8	73.4	74.4	72.9	69	70.8	71.3	68.4	64.2
	Koyonzo	JN053446	75.6	**98.6**	**92.2**	**90.5**	83.8	83.5	79.3	87.2	84.3	69.8	69.9	66.4	63.7
Siaya	Township	JN053454	75.2	**98.4**	**92.2**	**90.3**	84	83.6	79.6	87.2	84.1	69.7	69.6	66.7	63.9
	Nyamila	JN053455	75.2	**98.2**	**92**	**90.1**	84	83.6	79.5	87	84	69.7	69.6	66.7	63.9
	Komolo	JN053456*	75.8	**98.7**	**92.2**	**90.3**	84	83.7	79.4	87.3	84.3	69.8	69.9	66.7	63.8
	Luanda	JN053431*	**97.3**	75.1	71.8	71.3	74.9	73.5	74.5	73	69.1	70.9	71.4	68.5	64.3
	Luanda	JN053458*	74.9	**98.1**	**91.7**	89.7	83.7	83.2	79.3	86.5	83.6	69.3	69.4	66.2	63.2
	Oyombi	JN053426	**95**	73.4	70.3	70	73.4	72	73.1	71.7	67.7	70	70.2	67.5	63.1
	Oyombi	JN053429	**97.1**	74.9	71.7	71.1	74.7	73.4	74.4	73	68.9	70.8	71.2	68.5	64.1

Virus isolate* from cassava. Abbreviations and Genbank accession numbers are as given in Table 1. For ACMV [GenBank: NC001467.1] and EACMV-UG, [GenBank: NC004674.1] were used for comparison.
